# Relationship Between Item and Source Memory: Explanation of Connection-Strength Model

**DOI:** 10.3389/fpsyg.2021.691577

**Published:** 2021-09-29

**Authors:** Junjun Guo, Keith Shubeck, Xiangen Hu

**Affiliations:** ^1^School of Psychology, Central China Normal University, Wuhan, China; ^2^Department of Psychology, The University of Memphis, Memphis, TN, United States; ^3^Institute for Intelligent Systems, The University of Memphis, Memphis, TN, United States

**Keywords:** item, source, memory, connection-strength, model

## Abstract

The controversy in the relationship between item memory and source memory is a focus of episodic memory. Some studies show the trade-off between item memory and source memory, some show the consistency between them, and others show the independence between them. This review attempts to point out the connection-strength model, implying the different types and strengths of the important role of the item–source connections in the relationship between item memory and source memory, which is based on the same essence in the unified framework. The logic of the model is that when item memory and source memory share the same or relevant connection between item and source, they positively connect, or they are independently or negatively connected. This review integrates empirical evidence from the domains of cognition, cognitive neuroscience, and mathematical modeling to validate our hypothesis.

## Introduction

Effective retrieval cues play an important role in memory recovery. The effect of these cues is reflected in many research studies on memory: (1) directed forgetting paradigm (Sahakyan and Kelley, 2002); (2) mood-dependent paradigm (Lewis and Critchley, 2003); (3) emotional enhancement effects (Talmi et al., 2019); (4) false memory paradigm (Bookbinder and Brainerd, 2016), and (5) context maintenance and retrieval mathematical model to prove the importance of memory cues (Polyn et al., 2009). These phenomena reflect the implicit decision-making of memory based on effective cues that researchers call “sources” (Johnson et al., 1993), and these memory phenomena belong to declarative memory, which include episodic memory and semantic memory (Squire, 2004; Tulving, 2004).

Episodic memory and semantic memory are two different memory systems that were proposed by Tulving to cover human memory. Tulving believed that the differences between episodic and semantic memory are self-involvement, autonoetic awareness, and subjective sense of time (Tulving, 2004). Such features imply the specific attribute of episodic memory: connections. The differences between episodic and semantic memory are different types of connections. Researchers have confirmed the existence of a semantic network, which is called the spreading-activation theory (Collins and Loftus, 1975). Research shows that semantic memory also has connections, supported by connecting capacity in the hippocampus (Manns et al., 2003; Duff et al., 2020) and that patients with bilateral lesions have an impairment in semantic memory capacity relatively shortly before or after the damage has occurred but not in remote memory for factual knowledge. The relatively smaller damage to semantic memory, compared with episodic memory, in the hippocampus (Vargha-Khadem, 1997) is because semantic information is easier to connect to the semantic network, and this process is relatively automatic and unconscious. This idea will be discussed in section “Different Sources.” As a result, episodic memory and semantic memory form a large network, including items and sources, and memory is divided into two categories, item memory and source memory, based on attention allocation (which will be explained in detail in the next section). The former emphasizes the retrieval of an item based on implicit sources, while the latter emphasizes the explicit retrieval of sources.

Item memory usually explains a lot of phenomena including false memory (Reyna, 2000), working memory (Raaijmakers and Schiffrin, 1981), emotional memory (Talmi et al., 2019), and other forms of memory, such as recognition and recall (Johnson, 2005). These memories are all item memories based on “source information.” For example, time information is used as a source. Other research studies are focused on factors that affect the formation of source memory (for review, refer to Mather, 2007; Mitchell and Johnson, 2009). Relatively little attention has been paid to the relationship between item memory and source memory. The existing empirical evidence shows the dissociative (Glisky et al., 1995; Davachi et al., 2003; Slotnick et al., 2003), positive (Madan et al., 2017), or negative (Mather et al., 2006) relationship between item memory and source memory. Several research studies show that some factors have different effects on item memory and different source memory, such as attention and emotion (Mather, 2007).

However, there was a lack of a unified theoretical framework to explain the different relationships between item memory and source memory. We suggest that item memory and source memory share the same essence connections.

The connection-strength model emphasizes the important role of connections between item (semantic feature) and sources in the processes of encoding and retrieval of memory, and the relationship between the item and source memory, including positive, negative, or independent relationships.

Hence, we will form and introduce the connection-strength model to explain the relationships between item and source memory in the integrated framework, which is deeply based on item–source connections.

### Commonness Among the Different Kinds of “Memories” – Connections

In the study of memory, there are many different forms of memory. From the view of time, the memory includes “working memory,” “short-term memory,” and “long-term memory” and from the view of retrieval, the memory includes “item memory,” “source memory,” “context memory,” and “associative memory.” For Tulving (2002), memory can be divided into “episodic memory” and “semantic memory.” For Squire (2004), memory can be divided into “declarative memory” and “non-declarative memory.” Without a proper framework, these different types of memories seem to represent different connotations.

However, further reflection shows that these memories share different connections: “working memory,” “short-term memory,” and “long-term memory” depend on different item-temporal connections; “item memory,” “source memory,” and “associative memory” depend on similar or different item–sources connections, which will be elucidated in the following sections. “Declarative memory” and “non-declarative memory” are retrieved relatively intentionally or automatically based on connection, respectively.

From the view of Chalfonte and Johnson (1996), there is no essential or inherent difference between “item” and “source,” although they seem to be two different concepts. Such dichotomy comes from our attention focus: the focus of attention is called “item,” and other information is called “source.” For example, in psychological experiments, subjects always treat semantic features as the focus of our attention and treat other perceptual features as secondary information. Otherwise, only when the experimenters asked the subjects to focus on the secondary information, which researchers call source, the perceptual become “item” and “the semantic” become the new source. The “item” and “source” are two points of “connection” that are like a seesaw; sometimes one end is higher, and some other times, the other end is higher.

Consequently, memories are merely the associative network, and “episodic memory,” “semantic memory,” “source memory,” “associative memory,” and “context memory” are the subdivisions of such network. Different concepts underlie different endpoints in the connections. Source memory emphasizes retrieving source features, item memory emphasizes the retrieval of semantic features, and associative memory emphasizes the connection between different semantic features. Even emotions can be included in this network: mood-dependent memory (Lewis and Critchley, 2003). The view that emotion is merely one joint in our memory networks that tries to explain emotion-associated memory is called emotional priming.

**FIGURE 1:**
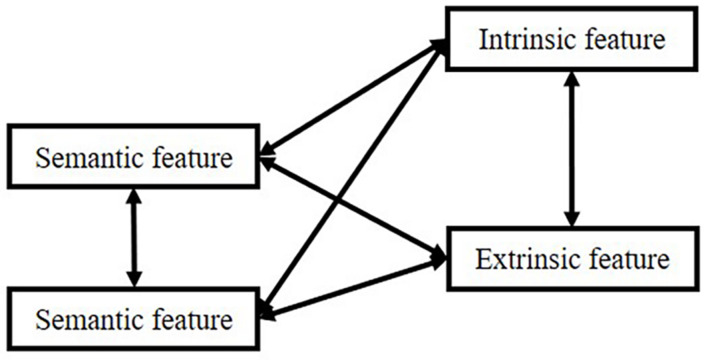
Interactions between items and sources.

However, there is an extensive concept or term confusion among these manifestations, such as “item” and “source”: sometimes, researchers equate “item” to “semantic meaning”; sometimes, “item” means the episodic concept, the association. Therefore, in the remaining part of the manuscript, we will use the connection: “item”-“source,” in which “item” means semantic features, and “source” means other information associated with “semantic.” However, in memory systems, “item” in “item memory” means the connection. We use these depictions because most researchers utilize these terms in such a manner. In this logic, all kinds of memory can be divided into two types based on our attention focus. The relationship is shown in Figure 1.

### Balance Between Item and Source Memory

From the perspective of the experiences of individuals, item memory and source memory are two parts of episodic memory, which are about the semantic and its association with subjective experiences, including when, where, and how an event happened. Source memories are results of questions that ask us to explicitly point out when, where, and how an event happened. The differences between item memory and source memory are as follows: the former tries to retrieve the item based on one kind or different kinds of sources, and the latter tries to retrieve one kind of source from the item. Hence, source memory and item memory are phenomena of connections.

From the perspective of the interaction between item memory and source memory, first, the memory of sources contributes to item memory; second, item memory also influences source memory. A wrong starting cue from the connection would impair true memory. This explains why the “feature-conjunction paradigm,” which shares features with presented items, would facilitate a false memory (Nie, 2018).

Recognition shows the forms of “recollection,” which is associated with the experience of vivid source attributes and “familiarity” without clear source memory (Yonelinas, 1999). Starns and Ksander (2016) used the zROC (the z-transformation of a operator characteristic curve which comes from the ratio between hit rate and false rate) slope to determine the relationship between item memory and source memory. They used three types of experimental conditions: (a) words associated with the source for once (no repetition); (b) words associated with two different kinds of sources (face or animal) (different source repetition); and (c) word and source connection repeats three times (same source repetition). Regardless of the condition, increased item memory confidence enhances the confidence of source memory, which may indicate the item as a cue to effectively retrieving the sources.

These examples reflect only a part of the scientific scenes we want to delineate. Johnson (2005) calls such phenomena “the different task shared the same processes.” However, few research has tried to understand the different memories in the unified framework, which only contain two types of memory: item memory and source memory, which combined episodic memory and semantic memory that are based on “connections.”

In various studies, there are always different relationships between item memory and source memory: (1) positive; (2) negative; and (3) irrelevant. However, there is a lack of integrated theories to explain such a controversy. Mather (2007) proposed an “object-based attention” framework to explain the better intrinsic source features memory and worse extrinsic source features memory for emotional stimuli. Nevertheless, this theory is insufficient to explain many inconsistent phenomena in memory. Sometimes, positive emotional events and positive context promote associative memory (Fredrickson and Branigan, 2005; Madan et al., 2019). However, sometimes, negative emotional context expands the scope of attention, and positive emotional context reduces the scope of attention (for review, refer to Huntsinger, 2013). In other studies, high or low motivational intensity related to emotion has different effects on the attention process (Harmon-Jones et al., 2011), and different emotions have different effects on attention and memory (Gable and Harmon-Jones, 2010; Harmon-Jones et al., 2011). These effects on attention will be reflected in the process of connection formation (which will be explained in section “Introduction of the Connection-Strength Model”).

We suggested that the different types and different strengths of connections between item and source, item and item, and source and source (these can be called item-source connections) play an important role in the relationship between item and source memory. In the next section, we presented the premises of the strength-connection model followed by the introduction of the connection-strength model. In the section “Evidence From Cognition, Cognitive Neuroscience, and Mathematical Models,” we collected evidence from domains of cognition, cognitive neuroscience, and mathematical cognitive psychology to validate our “strength-connection model” in interpreting the relationship between item and source memory.

## Premises of the Connection-Strength Model

Different kinds of “connections” exist in a memory system, which is the reason for different relationships between item memory and source memory. Factors that affect the formation and extraction of “connections” are as follows: different sources of natural existence, single source or combined source, formation of connections, and extraction of connections.

### Different Sources

There are always different source features that can be divided into different types. From the viewpoint of modality (Johnson et al., 1993), there are perceptual, contextual, semantic, and affective sources. From a relevant perspective, sources include the following: (1) external source monitoring, which demands us to discriminate different perceptions; (2) internal source monitoring, which involves two source discriminations in our thoughts; and (3) internal-external source monitoring, such as distinguishing source memory of thoughts from perception. The two classifications are hierarchical: the latter depends on the combination of the former.

From a spatial perspective, Mather (2007) tried to divide source features into two types: object-based source features, intrinsic features that share the same attention scope with the object, extrinsic source features that go beyond items such as context and associative objects. From the timeline, the source is presented before, after, and parallel with the item.

Bellezza and Elek (2018) illuminated that a bundle of two items and two sources in a unit affects different phenomena in memory. Bellezza and Elek presented word pairs with each word in 1 of 4 locations. In the test, subjects were shown one of the paired words and asked to recollect the other word and their locations. The results show that (1) the performance of source-location memory for the cue and the target is equal; (2) the source memory of unrecalled words is above chance; (3) the memory of the cue is associated with that of the target word; and (4) the location of the cue is always confused with that of the target. These results support the fact that item–source connections are always diverse and easy to change. A lot of different information will contribute to retrieval as an effective cue.

Other kinds of sources differ in the information process or extraction process: automatic or intentional encoding, automatic or deliberate retrieval, such as the temporal compared with the neutral environment, the semantic compared with the context. Based on our attention allocation, the process is automatic or strenuous. Divided attention deeply influences the deliberative process compared with the automatic process, either in encoding or retrieval.

Therefore, numerous connections are formed between and among “items” and “different sources.” These connections would also influence item memory (for example, Talmi et al., 2019) and source memory (for review, refer to Mather, 2007). Consequently, there are many possibilities for a cue to be called memory.

Formations of the connections are dependent on the three periods as follows.

### Three Periods of the Item–Source Formation

Although there are many different kinds of source features, three periods are involved in the formation of the item–source connections: “the perception of source features,” “the appraisal of the importance of source features,” and “connecting the item and source features.” These processes are all indispensable. The structure of item–source formation is demonstrated by the following logic based on relative importance: only when participants try to form a connection that the strength will be higher. The appraisal is the core of these processes.

The effects of adaptive memory and emotional memory can demonstrate the importance of these three processes: when sources that connect to the item are important, the sources will be memorized better. However, when the item is relatively more important than the source, the source memory will be worse memorized. These two processes both impair the formation of connections, because the focus only influences its connections that reflect the importance of appraisal. Kroneisen and Bell (2018) used survival-based or moving-based appraisals for the item and found that the source memory associated with survival processing is better than the other conditions. Consistent effects are found in the memory of the location of foods or potential predators after appraisals of ease of collecting food or capturing wild animals (Nairne et al., 2012). According to the same logic, when the item is important, such as in a contaminated environment (Fernandes et al., 2017), the hunter information for males or gathering information for females (Nairne et al., 2009), the memory will be better.

From the perspective of emotional memory, there is also a trade-off: the emotional material decreases the memory of neutral context (Kensinger et al., 2007; Mather, 2007), and emotional context impairs neutral item memory (Zhang et al., 2015). This emotional enhancement memory trade-off is based on personal goals (for review, refer to Levine and Edelstein, 2009). Relative importance influences appraisal, perception, and connection in a conscious or unconscious form.

Deep empirical evidence will follow in section “Introduction of the Connection-Strength Model.”

### Goal and Different Processes in Encoding and Retrieval

The encoding and retrieval processes are two important stages in memory. However, there are contradictions between the mechanisms of encoding and retrieval: is the relationship symmetric or asymmetric? Tulving et al. (1994) found that encoding and retrieval are asymmetrical in the hemispheric cortex. However, encoding and retrieval also share the hippocampus (Fritch et al., 2020; Guo and Yang, 2020).

From the viewpoint of the item–source connections, symmetric and asymmetric relationships depend on whether the encoding and retrieval processes share the same item–source connection and the time of detection in different experiments. Such connections may influence the activity of the prefrontal cortex, parietal cortex, and hippocampus. There are types of goal-oriented spatial learning that influence spatial encoding and retrieval in the hippocampus (Turi et al., 2019). Research has found that goals deeply influence human memory in terms of items, sources, and connections (for review, refer to Levine and Edelstein, 2009; Kaplan et al., 2012).

The goals come from two sources: different appraisals of different individuals, attentional locations, and demands of the experimental design. Occasionally, the two kinds of goals compete with each other, and the winner plays an important role in the formation of connection. For example, the emotional context before or after attention always changes attention and memory by motivation or goal (Kaplan et al., 2012). The goals between encoding and retrieval facilitate the common or different processes in item–source connections and memory, which are reflected in the hippocampus (Levita and Muzzio, 2010).

As a result, when encoding and retrieval are based on different connections, it is more difficult to retrieve the item or source. This is mainly derived from Formula (2) and Formula (3).

### Presentation of the Connection Models in Memory

The model originates from three existing popular theories: “spreading-activation theory” (Collins and Loftus, 1988), “searched for associative memory” (Raaijmakers and Schiffrin, 1981), and “hybrid model of source monitoring in paired-associates” (Bellezza and Elek, 2018). The description for these models is presented in Table 1.

**TABLE 1:** Old models that emphasize different connections in memory.

Theories	Authors and Cite	Emphasis	Example
Theory1 spreading-action theory	Collins and Loftus, 1975	Semantic features are connected networks.	Some semantic features are more closely related than others.
Theory2 hybrid model of source monitoring	Bellezza and Elek, 2018	The connections among items, sources exist.	One source of the item can be retrieved by the other item.
Theory3 search of associative memory	Raaijmakers and Schiffrin, 1981	Encoding in working memory; and importance of effective cues in retrieval.	In free recall, giving a cue-item always impairs the performance compared to no cued free recall, which means the importance of effective cue.

The spreading-activation theory (Collins and Loftus, 1988) emphasizes the connections among different concepts based on experiences of individuals and different connected strengths among them. When a concept is activated, the signal goes along the connection, and other concepts are activated. The stronger the connection, the easier it is to be activated.

The hybrid source monitoring model (Bellezza and Elek, 2018) attempts to explain the connections among items and their source features. Specific experiments are described in the first part. In the list, an item is not only associated with its source features but is also associated with other items and their sources. In the retrieval process, items and sources can also be used as cues to retrieve other items.

Raaijmakers and Schiffrin (1981) proposed a mathematical model to explain how a cue influences the performance of free recall. Such mathematical model is deeply embedded in the theory that retrieval and retrieval cues play an important role in episodic memory recovery, such as recognition and recall (Tulving and Thomson, 1973).

In encoding, connections are formed in working memory and come from the shared time in the capacity of working memory. Working memory cannot simultaneously maintain excessive information. Consequently, only the item and source share the same period in working memory, and connections can be formed.

**FIGURE 2:**
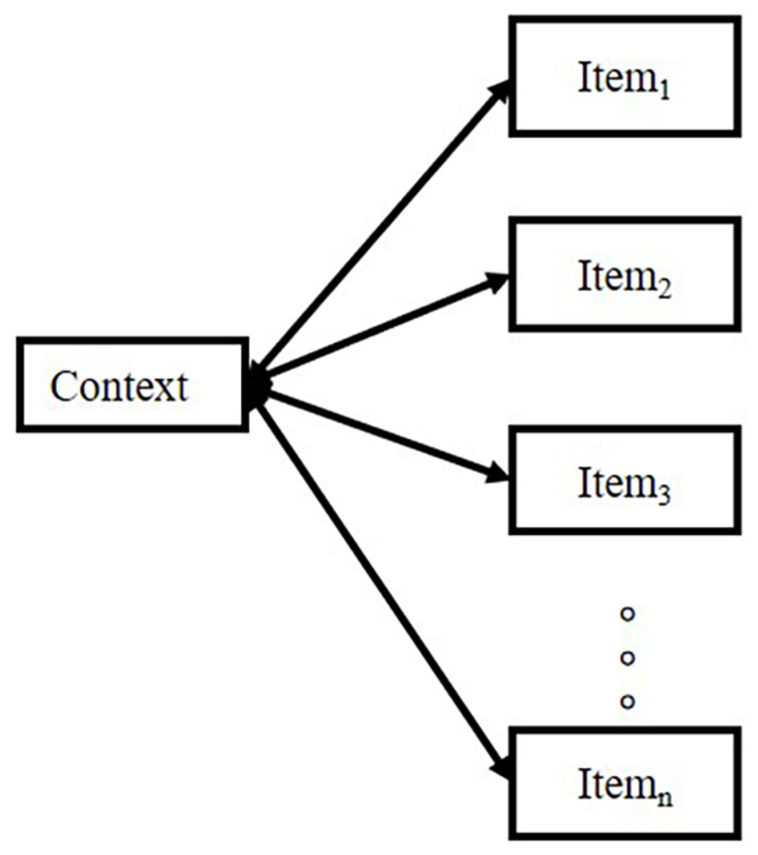
The logic of the model studied by Raaijmakers and Schiffrin (1981).

Therefore, an inappropriate retrieval cue presentation impairs the free recall of a list. For example, when asking subjects to recall a list by a cued item, the recall performance would be worse than the condition of free recall with no cue (Raaijmakers and Schiffrin, 1981). The model emphasizes logic, as illustrated in Figure 2.

Raaijmakers and Schiffrin (1981) pointed out that the probability of retrieving the item in the list depends on the strength ratio of “context-item” to “the sum of context-all other items strengths,” which can be simplified in the equation:


(1) $$P\,r\,o\,b\,a\,b\,i\,l\,i\,t\,y_{R}\,\left(i\,t\,e\,m_{i}\right)=\frac{s\,t\,r\,e\,n\,g\,t\,h_{\left(c\,o\,n\,t\,e\,x\,t_{i}-i\,t\,e\,m_{1}\right)}}{\left\{s\,t\,r\,e\,n\,g\,t\,h_{\left(c\,o\,n\,t\,e\,x\,t_{i}-i\,t\,e\,m_{1}\right)}+\ldots +s\,t\,r\,e\,n\,g\,t\,h_{\left(c\,o\,n\,t\,e\,x\,t_{i}-i\,t\,e\,m_{n}\right)}\right\}}$$

However, there are limitations in the model: (1) only other items in the list have been considered in whole connections; (2) concepts are ambiguous: “other items” is part of “context,” and the real context–temporal, semantic, and cognitive operations are not mentioned in the model; (3) such model is focused on the retrieval of item memory, not on source memory.

Three theories emphasize that information connection exists in our memory systems. In addition, effective retrieval cues are very important in memory recovery. However, these models cannot explain why there are differences between item and source memory, and why there are different relationships between the two memories.

## Introduction of the Connection-Strength Model

The item always connects with different kinds of information, including semantic information, perceptual infor- mation, contextual information (spatial or temporal information), affective information, and operative track. This information can be integrated into one cluster or one bundle, which we call “object.” The “object” involves a series of information that contributes to a whole representation. Although most of the time such integration cannot be realized by our consciousness, Kahneman et al. (1992) found that temporal proximity is very important in object-specific integration. Such capacity even happens in infants (Woodward, 1998). Attention plays an important role in object formation. Logan (1996) pointed out the two processes in the formation of an object: first, perceive the perceptual grouping from spatial proximity, and second, attention chooses several of them to form an object. Such theory is proved by a CODE theory–a mathematical model of visual attention. Attention always has a strong property of “selection” (for review, refer to Heinke and Humphreys, 2005), which is very important in the formation of connections.

As a result, many connections are constructed with a difference in strengths. Among these connections, only parts of them are to be used in item memory and source memory. An effective connection is a connection with higher strength. The main differences between item memory and source memory can be seen in the connections they call. The relatively significant difference is that connections in item memory are sometimes combinations of item-sources connections. The memory retrieval probabilities for items and sources are presented in Equations (2) and (3). However, connection for source memory is a specific connection, or may be mediated by other items.


(2) $$P\,r\,o\,b\,a\,b\,i\,l\,i\,t\,y_{R}\,\left(i\,t\,e\,m_{i}\right)=\frac{\left\{s\,t\,r\,e\,n\,g\,t\,h_{\left(i\,t\,e\,m_{i}-s\,o\,u\,r\,c\,e_{1}\right)}+\ldots +s\,t\,r\,e\,n\,g\,t\,h_{\left(i\,t\,e\,m_{i}-s\,o\,u\,r\,c\,e_{k}\right)}\right\}}{\left\{s\,t\,r\,e\,n\,g\,t\,h_{\left(i\,t\,e\,m_{i}-s\,o\,u\,r\,c\,e_{1}\right)}+\ldots +s\,t\,r\,e\,n\,g\,t\,h_{\left(i\,t\,e\,m_{i}-s\,o\,u\,r\,c\,e_{n}\right)}\right\}}$$

(3) $$P\,r\,o\,b\,a\,b\,i\,l\,i\,t\,y_{R}\,\left(s\,o\,u\,r\,c\,e_{i}\right)=\frac{s\,t\,r\,e\,n\,g\,t\,h_{\left(s\,o\,u\,r\,c\,e_{i}-i\,t\,e\,m_{i}\right)}}{\left\{s\,t\,r\,e\,n\,g\,t\,h_{\left(s\,o\,u\,r\,c\,e_{i}-s\,o\,u\,r\,c\,e_{1}\right)}+\ldots +s\,t\,r\,e\,n\,g\,t\,h_{\left(s\,o\,u\,r\,c\,e_{i}-s\,o\,u\,r\,c\,e_{n}\right)}\right\}}$$

*SI*_*i*_ means the employed source-item strength in source memory decision making, and $\sum_{t=1}^{n}S\,S_{t}$ means the sum of all the strengths of connections related to the “source”, whether the connection is formed in experiments or from past experiences. *P*_*R*_(*source*_*i*_) means the probability of a specific source retrieval.

**FIGURE 3:**
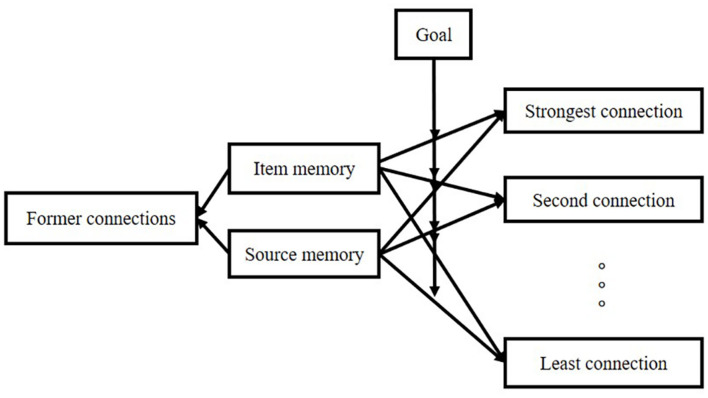
A rough description of the “connection-strength model.”

A rough description of the model is shown in Figure 3.

**FIGURE 4:**
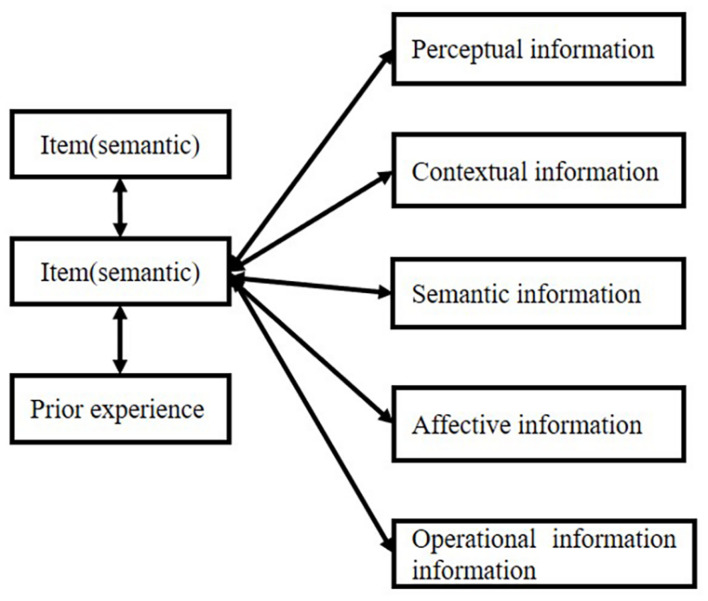
Connection-strength model in item retrieval.

Item retrieval follows these principles: (1) performance of item retrieval depends on the connection between item and source; (2) there are two kinds of connection: item-one-source connection and item-sources connection; (3) the higher the connection strength, the more likely it is to be called; (4) the connection strength comes from encoding period; (5) appraisal and attention are very important in the formation of connection-strength; (6) in item-sources connection, our different goal would assign a different weight to a different connection in a different experimental design. The structure is shown in Figure 4.

**FIGURE 5:**
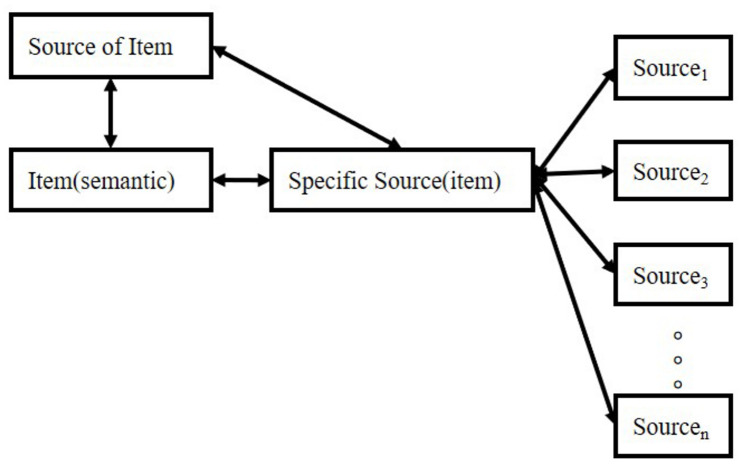
Retrieval of source memory.

Source retrieval follows these principles: (1) source retrieval depends on the ratio of a specific connection to the sum of all associated connections; (2) there are three types of connection: item-specific source connection, other item-the specific source connection, and other source-the specific source connection; (3) all the connections come from two stages: formation in the experiment and formation out of the experiment; (4) appraisal and attention are very important for the formation of connection-strength; (5) effective cues with higher strength are important for the performance of retrieval. The higher the connection strength, the more likely it is to be called. The structure is shown in Figure 5.

The essence of item memory and source memory is the same, which depends on the connection between item and source(s) and the ratio of the called connection(s) to the sum of all associated connections. The main difference is that focus shifts from item to source, and vice versa.

The relationship between item memory retrieval and source memory retrieval is: when the connection required by source memory and item memory is the same, there is a positive correlation between them; when item memory and source memory depend on different connections, for example, item memory depends on item-temporal connection, while source memory depends on item-color connection, item memory and source memory are independent of each other; when the connection required by item memory affects the formation of source memory connection, there is a negative correlation between item memory and source memory. These can be identified in the probability equation in equations (2) and (3): when the numerator of the ratio increases at the same time, the positive relationship appears, otherwise, the negative relationship will appear. When the numerator is irrelevant to each other, they are independent.

There is a lot of evidence to support our model: first, the performance of source memory is always worse than that of item memory, because the connection required by the source is a part of the connections required by item memory, such as affective state as the source; second, several source memories are better than others because they call stronger connections, such as object-based colors, compared with other associated objects; third, attention plays an important role in connection formation, the more attention paid to the connection, the higher the strength of the connection; fourth, experimental design affects which connection will be encoded and called by item memory and source memory; fifth, mathematical models show that both item memory and source memory are continuous processes, which indicates that the different connection-strength deeply affects the performance of item memory and source memory. A mathematical model also shows that the confidence of item memory and source memory influences each other (Starns et al., 2013).

The empirical evidence supporting these phenomena is as follows.

### Different Experimental Designs

The two types of experimental designs are considerably different. First, studies presenting recognition and source judgments for the same item in immediate succession have revealed chance-level accuracy in source memory with no recognition. Second, studies presenting a block of recognition followed by a block of source judgments have revealed above-chance accuracy in source memory with no recognition (for review, refer to Fox and Osth, 2020).

The essence behind these phenomena is that subjects call different connections. When source judgments occur in the block after all the recognition has finished, other items associated with unrecognized items will also activate the source without recognition. Fox and Osth (2020) used a simultaneous, blocked, and reversed blocked design to demonstrate this idea.

Kim et al. (2012) found negative effects of item repetition on source memory. Experimenters show the items in two phases. In phase 1, line drawings present varying numbers of items; in phase 2, each item is associated with a critical new source. The results show that the more repetition in phase 1, the more difficult it is to memorize the critical new source in phase 2. This can be explained by the connection-strength model. The more repetition of the items in list 1, the more item–source (temporal source) in list 1, which induces a relatively weaker item-critical new sources strength ratio. Osth et al. (2018) found a list-strength effect, which means a proportion of items are strengthened to observe the effect on non-strengthened items in source memory but not in item memory. This effect can also be explained by our model.

## Evidence From Cognition, Cognitive Neuroscience, and Mathematical Models

Empirical evidence supports the connection-strength model, including evidence from cognition, cognitive neuroscience, and mathematical models.

### Evidence in Cognition

#### Attention in Unitization

**FIGURE 6:**
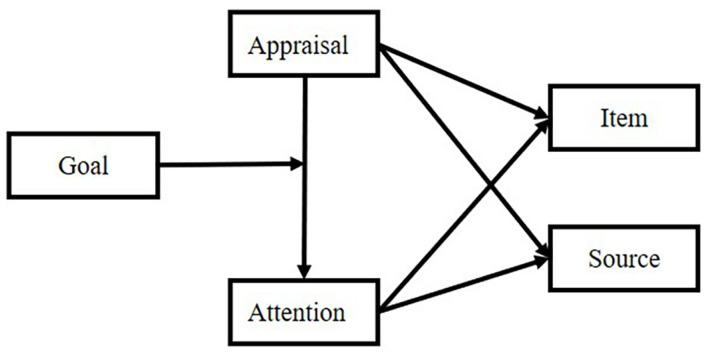
Attention interaction between item and source.

Attention plays the most important role in forming connections (for review, refer to Mather, 2007; Block and Gruber, 2014). The relationship is shown in Figure 6.

The object-based attention model (Mather, 2007) and space-based attention model (Belardinelli, 2016) support the importance of attention in the connection of different features in an object or space. However, not all connections are formed and stored in memory; for example, intrinsic source feature memory is always better than extrinsic source feature memory. This is because the intrinsic source features of the object share scope of attention with the object, which makes it easier to form a connection. These relationships are reflected in emotional enhancement memory (for review, refer to Talmi et al., 2019) and enhanced intrinsic source memory (for review, refer to Mather, 2007). Simultaneously, memories of extrinsic source features are impaired because they are beyond the core of attention.

Thus, the first evidence in cognition comes from the operation of attention to enhance the connection between the item and the source. The difficulty level of unitization deeply affects the connection between the item and the source, and several factors affect the difficulty: “pre-experimental associations” (e.g., Giovanello et al., 2006; Rhodes and Donaldson, 2008; Ford et al., 2010), “experimentally instructed encoding strategy” (Haskins et al., 2008; Bader et al., 2010; Parks and Yonelinas, 2015), and characteristics of the source. These factors are related to the same concept—unitization. The former and the latter reduce the difficulty of attention integration; the second is used as an effective strategy to enhance connection formation.

In research, the performance of aging declines in episodic memory (Friedman, 2013), semantic memory (Bertola et al., 2019; Venneri et al., 2019), source memory (Schacter et al., 1991), and associative memory (Greene and Naveh-Benjamin, 2020). Researchers suggest that these phenomena arise from a reduced binding capacity (Li et al., 2005). Unitization can reduce this tendency. Zheng et al. (2016) used two conditions: unitized condition (imaging the color as the internal parts of item) and non-unitized condition (imaging the color as the context), and then asked subjects to complete the source memory test. The results show that the difference in source memory between young and old people is smaller under the condition of unitization. Boywitt and Meiser (2012) found that under the condition of incidental attention, the source-source connection disappears in the extrinsic source features; and that this connection is preserved in intentional attention.

Further research (Kinjo, 2011) shows that the damage of extrinsic source memory is greater than that of intrinsic source memory because the former needs more attention.

#### Emotional Memory Enhancement Effects

The formation of connection plays an important role in emotional reinforcement memory, which is based on two different kinds of connection, what researchers call “organization” and “emotional context.” Emotion enhances item–item connection and item–source connection. When emotional materials are highly clustering with each other, memory is always better than low-clustering materials (Talmi et al., 2007). Talmi and Moscovitch (2004) found that semantic relatedness was considerably important in list item memory: the memory of semantic-related neutral words is not worse than that of emotional words. Talmi et al. (2019) set up a retrieved-context model to explain emotional enhancement in memory. The emotional context maintenance and retrieval model points out that the emotional enhancement memory effect is based on the enhanced item-source connection that is associated with ever-changing temporal and emotional context. Consequently, the cues of organization and context play an important role in emotion-enhanced effects.

#### Interaction Between Item Memory and Source Memory

For intrinsic source features, item memory is positively correlated with source memory. This is because the intrinsic source features share an attention system with the item. Therefore, the enhancement of item memory is positively related to the enhancement of intrinsic source memory, which is deeply reflected in emotional items (Mather, 2007).

The important trade-off between the item and extrinsic source is very important for connection formation. Attention to the item enhances the connections associated with “this item,” such as the temporal and color, rather than extrinsic sources (e.g., other items) that are beyond the item. Attention to “source” transfers the “source” to “item” and facilitates connections for the “new item.” The new connections increase the denominator of the probability formula, thus reducing the ratio of the item to the extrinsic source. Therefore, there is a negative relationship between item memory and source memory. Such phenomena are more deeply reflected in the memory related to emotion.

Source memory can be divided into two types: intrinsic source memory, which is the features of the item itself, and extrinsic source memory, which is the associated features outside the item, including the context and objects that are associated with the item. Numerous studies support two opposite phenomena: compared with neutral items, emotional items always facilitate intrinsic source memory; however, emotional items always interfere with extrinsic source memory.

The trade-off between emotional items and emotional source memory validates our theory. The first evidence comes from the relative importance of the item. Compared with positive emotional stimuli, the extrinsic source memory of negative emotional stimuli is worse (Madan et al., 2019). Compared with low-arousal emotional stimuli, the extrinsic source memory of higher arousal stimuli is worse (Mather et al., 2006; Kensinger et al., 2007; Mather, 2007). The second evidence comes from the relative importance of the source. Compared with a neutral context, an emotional context is always remembered better at the cost of neutral items (Maratos et al., 2001; Maratos and Rugg, 2011; Chiu et al., 2013). We are always attracted by the emotional context even when asked to keep the attention on items, especially in an extremely important environment, such as the source memory of cheaters (Kroneisen and Bell, 2013) and goal-inconsistency phenomena (Bell et al., 2012). The third evidence comes from a relative comparison between items and sources. When an item is emotional, item memory is better at the cost of neutral context (Kensinger et al., 2007), and researchers ask participants to remember words in the emotional context and find the impaired word memory influenced by the emotional context (Zhang et al., 2015). The fourth evidence comes from the reappraisal that can change the trade-off memory between items and sources. Steinberger et al. (2011) found that different reappraisals of items and contexts would facilitate different memory trade-offs.

### Evidence From Cognitive Neuroscience

**FIGURE 7:**
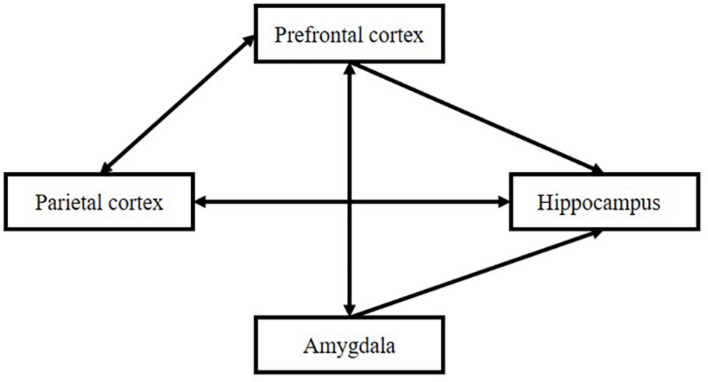
Interactions among subregions of the brain.

The formation of the item–source strength depends on three periods, “perception,” “evaluation,” and “association,” which can be reflected in the function of the brain: the parietal cortex, prefrontal cortex, and hippocampus. The hippocampus is the core structure in Tulving’s episodic memory. Its main function is to connect different types of information. At the same time, it is affected by different brain regions, such as the parietal cortex, frontal cortex, and amygdala. The relationship is shown in Figure 7.

#### Parietal Cortex in Perception

Only when a stimulus is perceived can a connection be formed. The posterior parietal cortex plays an important role in perception, such as speech, visual motion (Buchsbaum et al., 2010), and tactile perception (Ro et al., 2004). Transcranial magnetic stimulation of the right parietal lobe disrupts the perception of briefly presented stimuli (Howard et al., 2019). Damage to the intraparietal cortex impairs action and perception (Medina et al., 2020).

The parietal cortex is always a bridge between perception, action, and cognition (Gottlieb, 2007), which is associated with spatial attention and the “salience representation” of the external world that is relevant to us. The parietal cortex also combines with the amygdala to perceive biological motions (Bonda et al., 1996). Recently, researchers found that the parietal cortex plays an important role in transsaccadic memory and the integration of visual object features (Dunkley et al., 2016).

Extensive research has shown that cathodal stimulation on the left posterior parietal cortex decreases the retrieval performance in source memory (Chen et al., 2016. Guidotti et al. (2019) found that predictive activity in the parietal cortex predicts source-memory decisions: the greater the decision evidence, the greater the activation in the parietal cortex. The posterior eye field (Müri et al., 1996) and the ratio of posterior-anterior medial temporal lobe volumes can predict the performance of source memory (Snytte et al., 2020). Transcranial direct current stimulation of the parietal cortex decreases false recognition increases item and source memory accuracy compared with the situation with no stimulation (Pergollzzi and Chua, 2017), and improves associative memory (Vuli et al., 2021). The activation of the parietal cortex is associated with our confidence in memory: the activation of the old is larger than the new, and the perceived is larger than the imagined (King and Miller, 2017). Functional MRI (fMRI) shows parietal cortex plays an important role for information connections, especially in the object (von Stein et al., 1999), and lesions in parietal cortex will eliminate this processing (Decoteau and Kesner, 1998). Ben-Zvi et al. (2015) identified that parietal lesions impair associated learning, including word pairs, picture pairs, and picture–sound pairs.

#### Prefrontal Cortex in Evaluation

Classic research (Buschman and Miller, 2007) illustrates the role of the prefrontal cortex in the activation of posterior parietal cortices for top–down and bottom–up attention, which supports the idea that attention is the momentary enhanced reaction potential of the perceptual response (Berlyne, 1951). The low-frequency synchrony between the frontal and parietal cortices reflects top–down attention, and higher frequencies are associated with bottom–up attention. The prefrontal cortex plays an important role in the behavioral approach and inhibition processes (Sutton and Davidson, 1997), which is supported by observations of an activation of a “hot spot” at the cost of lateral inhibition through the call of norepinephrine (Mather et al., 2016). The interaction between the prefrontal cortext and parietal cortext is essential in the process of inhibition and activation (Buschman and Miller, 2007).

The prefrontal cortex has two main functions: “appraisal” and “attention allocation.” The appraisal is reflected in the situation, which reflects the evaluation of importance among different kinds of information. Researchers call this effect the “appraisal-by-content model” (Dixon et al., 2017). The model points out that different areas of the prefrontal cortex are responsible for different kinds of input, including outside perceptions, episodic memories, future events, viscera sensory, action, and emotions. Based on the appraisal, the prefrontal cortex decides where attention should be located.

This effect is reflected in the processing of emotional stimuli. In the study, “A cognitive-motivational analysis of anxiety” (Mogg and Bradley, 1998) “appraisal” of the events plays an important role in negative attention bias in the population of “anxiety.” Anxiety always shows a higher threat appraisal of negative stimuli than a healthy population. As a result, people with anxiety attempt to focus on negative stimuli because of a high threat appraisal of the negative stimuli; However, non-anxious groups think that negative stimuli do not pose a threat, and consequently they don’t pay attention to them, but continue to do what they are doing (Huntsinger, 2013). Many studies support appraisal bias in attention and memory (Ma et al., 2017; Foley, 2018). An fMRI shows that the prefrontal cortex in anxiety controls attention to threat-related stimuli (Bishop et al., 2004).

Increasing research supports the function of appraisal in the prefrontal cortex. Kalisch et al. (2006) found that the medial prefrontal cortex plays an important role in the high-level appraisal of emotional materials. The decreased function of the medial prefrontal cortex in Alzheimer’s patients will reduce their evaluation of their cognitive ability, especially memory (Ries et al., 2012). An explanation of the aversive would modulate the activation of appraisal in the medial prefrontal cortex (Mechias et al., 2009).

The prefrontal cortex plays an important role in the formation of connections. Research has found that normal aging and prefrontal cortex lesions are associated with poor performance in item memory and source memory (Swick et al., 2006). The activation of the medial prefrontal cortex contributes to the item and source memory of self-referent information compared with other referent information, which also reflects the role of appraisal (Leshikar and Duarte, 2012). Furthermore, prefrontal deficits impair episodic memory in patients with schizophrenia (Ragland et al., 2009). The subregions of the prefrontal cortex, left frontopolar cortex, left mid-ventrolateral region, left mid-dorsolateral region, and anterior cingulate cortex contribute commonly to working memory, semantic memory, and episodic memory (Nyberg et al., 2003).

#### Hippocampus in Association

The hippocampus is significant in connecting items and their sources. Dalton et al. (2018) used fMRI and found that the hippocampus cooperates with other areas to support the associative processes and scene constructions, which imply the binding ability of the hippocampus. Further research has found that the hippocampus is particularly important for the building of association across stimulus domains, such as combining visual features with auditory features (Borders et al., 2017). Implicit associative learning engages the hippocampus and interacts with explicit associative learning (Degonda et al., 2005).

Nordin et al. (2017) compared the performance of associative memory with the volume of the anterior hippocampus between middle-aged and older patients. The results show that the older population has poorer associative memory, which is accompanied by a smaller volume of the anterior hippocampus and less activation compared with the younger population. Iwasaki et al. (2021) found that beta oscillations in the hippocampus could forecast the performance of object-location associative memory. In mice, an increase in beta oscillations in the hippocampus during the encoding process would come along with better “source memory.” The hippocampus of primates and humans contains spatial view neurons, which provide a representation of locations in the viewed space. Neuronal networks in the hippocampus activate together to form episodic memory, especially recent events that involve relations (Giovanello et al., 2010). Gradual changes in hippocampal activity are crucial in remembering the order of events (Manns et al., 2007).

Other studies have shown that the activation of the amygdala due to emotional stimuli processing impairs the connection between emotion items and source features by disrupting the function of the hippocampus (Roozendaal et al., 2009; Madan et al., 2017).

#### Association Among the Parietal Cortex, Prefrontal Cortex, and Hippocampus

The prefrontal hippocampus circuit is significant in associative memory. Different units are activated by different item presentations in the prefrontal cortex and hippocampus according to time for monkeys. The research demonstrates that both the prefrontal cortex and the hippocampus contribute to feature binding according to the timeline (Cruzado et al., 2020). Interactions between the prefrontal cortex and the hippocampus are particularly important for reactivating memories and their contexts to contribute to memory retrieval and assimilate the new memories-item-source connection to our schemas (Preston and Eichenbaum, 2013). The recovery of extinct fear memory in a special context requires both the prefrontal cortex and the hippocampus (Milad et al., 2007). Prefrontal hippocampal interactions are obvious during the encoding of new memories (Takehara-Nishiuchi, 2020). Two processes may exist in such interactions: first, the formation of new information into the old memory networks; and second, the formation of different types of information into unification. For the process of retrieval, prefrontal-hippocampal interaction is also found in rats when rats try to decide where they should go inside a maze (for humans, refer to Öztekin et al., 2009; for animals, refer to Cholvin et al., 2016). Attention from the prefrontal cortex influences the activation of the hippocampus (Córdova et al., 2019).

Prefrontal-parietal connections are also particularly important. Bor and Seth (2012) pointed out the significance of the prefrontal-parietal network in attention, working memory, and chunking. The prefrontal cortex influences the parietal cortex by enhancing attention to specific perceptions involved in source feature processing (Katsuki et al., 2015; Sofia and Gregoriou, 2017).

Amygdala’s activation decreases or enhances the function of hippocampus (Madan et al., 2017) and interaction between parietal cortex and amygdala influences association forming (Kesner, 2000).

The research also found the combined contribution of the prefrontal cortex, parietal cortex, and hippocampus to working memory retrieval (Öztekin et al., 2009).

The different activation of areas that come along with source memory and episodic memory in neural imaging and neural physiology depends on the time course of detection. When detection time occurs simultaneously in one of the three proposed periods, perception, evaluation, and binding (association), the results would show that the activation of different areas in the brain supports episodic or source memory.

### Evidence From Mathematical Models

Several mathematical models attempt to explain the relationship between item memory and source memory. To answer this question, two important questions must be clarified. First, how is the experiment designed? Second, what processes are included in item memory or source memory? The different experiment designs and “processes” will influence the relationship between the item and source memory, which is based on the same or different connections in the model that we propose.

These models include the “multinomial processing tree,” “receiver operating characteristic analysis,” “context maintenance and retrieval model,” and “bivariate signal detection model.” However, a detailed description of the mathematical theory is beyond the scope of this article. We merely concentrate on how the mathematical models influence our knowledge of understanding the “connection-strength” model and validate our theories.

Contrary to the abovementioned evidence, mathematical models focus on retrieval processing in item and source memory. The essence that is for both encoding and retrieval is “item–source connection,” where encoding is trying to form the connection and retrieval is trying to recover the “connection” with a cue or cues. The following shows the understanding of the relationship by deploying the connection in our retrieval.

#### Multinomial Processing Tree for Source Memory

Batchelder and Riefer (1990) used the multinomial processing model to explain the phenomena of source monitoring and presented item detection, source identification, and guessing bias parameters. The model is based on the hypothesis that the retrieval of source memory only occurs when an item is detected. Bell et al. (2016) supported this hypothesis: in retrieval, items and distractors are randomly present, and researchers first ask subjects to make the recognition followed by source decision. The results showed no source memory with no recognition. Additionally, source memory is always worse than item memory. Such phenomena come from the experimental design-simultaneous presentation described in section “Introduction,” which cannot be treated as a theory but as the hypothesis rooted in the experimental design. The advanced multidimensional source model (Meiser and Bröder, 2002) shows that item memories are associated with source detection memories. Recollection is always associated with the joint memory of different source attributes, such as color and position; however, different sources are independent of each other in the condition of familiarity.

The multinomial processing tree model also supports different types of item–source connections with different strengths. Not all sources can be included in an item-source network (Dodson et al., 1998; Klauer and Wegener, 1998). Source memories of pictures are always better than visual words, and the source memory of the self-referent is better than other-referent information (Riefer et al., 1994). Meiser and Bröder (2002) showed different degrees of difficulty in source monitoring: when sources are similar, discrimination between sources is difficult.

#### Receiver Operating Characteristic (ROC) Analysis

Receiver operating characteristic curves are used as indices of whether the memory processes are based on the threshold criteria. The basic procedures behind the ROC are the first subjects to decide on the memory and then tell their confidence.

This topic focuses on three themes: whether two kinds of memory, “recollection” and “familiarity,” share the same process; whether the item memory and source memory share the same process; and how item memory and source memory influence each other.

Some studies state that “recollection” and “familiarity” share different processes: “recollection” is a threshold process, and “familiarity” is a graded process. Jacoby (1991) identified that “recollection” was deeply influenced by attention compared with “familiarity,” and had the same effect on older adults (Jacoby et al., 2005) and individuals with amnesia (Kensinger and Corkin, 2008). However, the main evidence comes only from neuroscience. This is because connections in “recollection” are more difficult to form and more easily influenced by other factors. From the viewpoint of source monitoring, researchers believe that the two processes are both based on the graded experiences of the subjects in asssociation with combined source information.

An excellent model, which is computational and based on neurobiology, was deployed by Elfman et al. (2008). The research found that the features of sources play a significant role: recollection will fit a threshold model when sources are considerably distinct and a continuous model when there is similarity (feature overlap) in sources. Such research finds that different activation of the hippocampus is extremely crucial in the encoding of different kinds of memory: distinct sources are associated with higher activity in the hippocampus, and lower distinct sources are associated with lower activity in the hippocampus. This classic research may solve the conflict in theory by the different activation in the hippocampus, which implies continuous item memory regardless of recollection or familiarity. The use of different source materials (In these types of experiments, variables such as pictures, words, varying colors, and auditory input are all significant predictors of forms in operator characteristic curves) in the experiments is significant in ROC analysis. In the experiment, Slotnick (2010) presented objects on the left or right position of the screen and asked subjects to remember. Recollection-based ROCs are formed by source memory confidence ratings connecting to judge “remember” or the highest item confidence rating response. The results of the ROCs show that recollection-based ROCs identify the hypothesis of continuous models. This evidence shows that the recollection and familiarity of recognition both follow continuous processing. Onyper et al. (2010) used the new model, “some-or-none,” to emphasize the importance of the continuous model. This is because the researchers cannot integrate the data from “words” and “travel scenes” by the dual-process signal detection theory or unequal-variance signal detection model (the threshold or continuous model). Researchers combine the dual-process model and continuous model into the “variable-recollection dual-process model,” which suggests that familiarity and recollection are based on the continuous process. However, the difference between familiarity and recollection is a variable criterion. The above studies mainly focus on how source memory affects item memory. Starns et al. (2013) used zROC slopes to explore the relationship between item memory and source memory. The experimenters presented the item with different sources, female or male voice (strong voice-presenting four times; weak voice-presenting one time), for different times and then asked the subjects how many times the item was present and what confidence they believed for the source. The results indicate that the confidence of item memory strengthens the confidence of source memory, which is called “the converging criteria account.” The essence is underlain by the experimental design: the repetition to present item, the source would simultaneously increase the strength of item–source connections.

For the question of whether the retrieval of item memory and source memory shares the same process, researchers answer this question. Slotnick and Dodson (2005) found that recognition and source memory are both continuous by removing non-diagnostic source information in the analysis. In the experiment, 160 words were presented by female or male voices and then were combined with 80 new words to ask subjects to evaluate the confidence of the item and source memory. This supports the fact that model-item memory and source memory share the same mechanism.

#### Context Maintenance and Retrieval Model: How Context Influences Item Memory

Howard and Kahana (2002) focused on how context influences item memory. Howard and Kahana (2002) first pointed out the temporal context model (TCM) to explain the well-known phenomena in human memory: the recency effect and contiguity effect. TCM considers the temporal context as the cue to retrieve the item; for the recency effect, the slightly changed item-retrieval temporal context, compared with the encoding context, contributes to recency, and the similar temporal context between continuous items supports the contiguity effect. Second, Sederberg et al. (2008) further evidenced the context-based theory of recency and contiguity by simulating the internal contextual state as an effective cue to retrieval that goes beyond the information of the time, which comes from a combination of different contextual information. Polyn et al. (2009) pointed out in context maintenance and retrieval model: the semantic source which comes from longstanding semantic association among words, the temporal source which reflects the presentation of sequences and the modality source which is the presentation form, all contribute to item retrieval. The interaction among the three parts jointly contributes to retrieval as an effective cue in free recall. This model can also explain the enhanced emotional memory by increasing the connection between emotional items and contexts (Talmi et al., 2019).

#### Bivariate Signal Detection Model

The “bivariate signal detection model” is an effective mathematical model for identifying the connection-strength model, which underlines existing different item–source connections. The “bivariate signal detection model,” as the special case of “multidimensional signal detection theory,” is the extension of the signal detection theory. The model considers that the mental state is large and noisy, and every action needs a judgment, and judgments can be modeled as a random sample from a multivariate probability distribution that reflects individual perceptual space. Decision-making always depends on the different axis mapping from the space in distribution.

Banks (2010) proposed that item memory and source memory share a single analytic model. The multidimensional signal detection theory states that recognition memory and source memory depend on the projection of the multidimensional configuration onto an appropriate unidimensional axis, which is deployed as evidence to make memory decisions. This explanation is consistent with our strength model, which implies that source memory and recognition share the same or different item–source connections; however, they are based on the same mechanism. Researchers have used mathematical model bivariate signal detection to demonstrate this hypothesis. The experiment presents two kinds of words (words and first names) along with two kinds of sources (visual and auditory). Subsequently, the displayed items and new items are presented on the screen, and subjects should give the confidence of the item and source memory. The results, which are analyzed in bivariate signal detection theory, show that different tasks (recognition, source memory) can be performed based on different decision axes from the projection of multidimensional configuration. In addition, the results show the orthogonal relationship between item recognition memory and source memory, which deploys different connections. However, recognition and source memory use the same memory database that employs different information connections.

## Conclusion

According to Johnson, memory can be divided into two types: information in the focus of our attention and information out of the focus of our attention. This dichotomy integrates a memory system into a new perspective, dividing it into item memory and source memory.

Different item memory and source memory depend on the same or different item–source connections. As a result, the relationship between item and source memory is positive, negative, or irrelevant.

Different item-source calls depend on the strength of the item-source(s) connection, which means that when the connection between item and source is stronger, the probability of retrieval of item memory or source memory is greater. However, when goals are proposed, different weights are added to different item-source connections; the memory then changes.

In different environments, there are different goals that affect the choice of connections. This explains why item memory and source memory are either consistent or inconsistent.

Cognitive processes and brain mechanisms affect the formation and intensity of connections. From the perspective of the cognitive process, attention allocation and the appraisal of the importance of items and sources would influence the formation of the item–source connection. From the perspective of brain mechanisms, the prefrontal cortex, parietal cortex, and hippocampus are associated with perception, appraisal, and connection formation, respectively. From the perspective of experimental design, the single source memory decision-making after item-random presents or block-items present determines the relationship between the item and source memory. The mathematical models support the hypothesis of the connection-strength model.

## Data Availability Statement

The raw data supporting the conclusions of this article will be made available by the authors, without undue reservation.

## Author Contributions

JG was responsible for the title selection, literature collection, and writing. XH proposed the suggestions for title selection and manuscript writing. KS provided input and feedback and helped with writing. All authors contributed to the article and approved the submitted version.

## Author Disclaimer

The content is solely the responsibility of the authors and does not necessarily represent the official views of NSFC.

## Conflict of Interest

The authors declare that the research was conducted in the absence of any commercial or financial relationships that could be construed as a potential conflict of interest.

## Publisher’s Note

All claims expressed in this article are solely those of the authors and do not necessarily represent those of their affiliated organizations, or those of the publisher, the editors and the reviewers. Any product that may be evaluated in this article, or claim that may be made by its manufacturer, is not guaranteed or endorsed by the publisher.

## References

[B1] Bader R. Mecklinger A. Hoppstadter M. Meyer P. (2010). Recognition memory for one-trial-unitized word pairs: evidence from event-related potentials. *NeuroImage* 50 772–781. 10.1016/j.neuroimage.2009.12.100 20045471

[B2] Banks W. P. (2010). Recognition and source memory as multivariate decision processes. *Psychol. Sci.* 11 267–273. 10.1111/1467-9280.00254 11273383

[B3] Batchelder W. H. Riefer D. M. (1990). Multinomial processing models of source monitoring. *Psychol. Rev.* 97 548–564. 10.1037/0033-295X.97.4.548

[B4] Belardinelli A. (2016). *Object-Based Attention: Cognitive and Computational Perspectives. From Human Attention to Computational Attention.* New York, NY: Springer New York.

[B5] Bell R. Buchner A. Kroneisen M. Giang T. (2012). On the flexibility of social source memory: a test of the emotional incongruity hypothesis. *J. Exp. Psychol. Learn. Memory Cogn.* 38 1512–1529. 10.1037/a0028219 22545603

[B6] Bell R. Mieth L. Buchner A. (2016). Emotional memory: no source memory without old-new recognition. *Emotion* 17 120–130. 10.1037/emo0000211 27504597

[B7] Bellezza F. S. Elek J. K. (2018). A hybrid model of source monitoring in paired-associates learning. *J. Exp. Psychol. Learn. Memory Cogn.* 45 1042–1065. 10.1037/xlm0000639 30024263

[B8] Ben-Zvi S. Soroker N. Levy D. A. (2015). Parietal lesion effects on cued recall following pair associate learning. *Neuropsychologia* 73 176–194. 10.1016/j.neuropsychologia.2015.05.009 25998492

[B9] Berlyne D. E. (1951). Attention, perception and behavior theory. *Psychol. Rev.* 58 137–146. 10.1037/h0058364 14834296

[B10] Bertola L. Ávila, Rafaela T. Bicalho M. Malloy-Diniz L. F. (2019). Semantic memory, but not education or intelligence, moderates cognitive aging: a cross-sectional study. *Braz. J. Psychiatry* 41 535–539. 10.1590/1516-4446-2018-0290 30994856 PMC6899367

[B11] Bishop S. Duncan J. Brett M. Lawrence A. D. (2004). Prefrontal cortical function and anxiety: controlling attention to threat-related stimuli. *Nat. Neurosci.* 7 184–188. 10.1038/nn1173 14703573

[B12] Block R. A. Gruber R. P. (2014). Time perception, attention, and memory: a selective review. *Acta Psychol.* 149 129–133. 10.1016/j.actpsy.2013.11.003 24365036

[B13] Bonda E. Petrides M. Ostry D. Evans A. (1996). Specific involvement of human parietal systems and the amygdala in the perception of biological motion. *J. Neurosci.* 16 3737–3744. 10.1523/jneurosci.16-11-03737.1996 8642416 PMC6578830

[B14] Bookbinder S. H. Brainerd C. J. (2016). Emotion and false memory: the context-content paradox. *Psychol. Bull.* 142 1315–1351. 10.1037/bul0000077 27748610

[B15] Bor D. Seth A. K. (2012). Consciousness and the prefrontal parietal network: insights from attention, working memory, and chunking. *Front. Psychol.* 3:63. 10.3389/fpsyg.2012.00063 22416238 PMC3298966

[B16] Borders A. A. Aly M. Parks C. M. Yonelinas A. P. (2017). The hippocampus is particularly important for building associations across stimulus domains. *Neuropsychologia* 99 335–342. 10.1016/j.neuropsychologia.2017.03.032 28377162 PMC5493148

[B17] Boywitt C. D. Meiser T. (2012). The role of attention for context–context binding of intrinsic and extrinsic features. *J. Exp. Psychol. Learn. Memory Cogn.* 38 1099–1107. 10.1037/a0026988 22250914

[B18] Buchsbaum B. R. Hickok G. Humphries C. (2010). Role of left posterior superior temporal gyrus in phonological processing for speech perception and production. *Cogn. Sci.* 25 663–678. 10.1207/s15516709cog2505_2

[B19] Buschman T. J. Miller E. K. (2007). Top-down versus bottom-up control of attention in the prefrontal and posterior parietal cortices. *Science* 315 1860–1862. 10.1126/science.1138071 17395832

[B20] Chalfonte B. L. Johnson M. K. (1996). Feature memory and binding in young and older adults. *Memory Cogn.* 24 403–416. 10.3758/BF03200930 8757490

[B21] Chen N. F. Lo C. M. Liu T. L. Juan C. H. Muggleton N. G. Cheng S. K. (2016). Source memory performance is modulated by transcranial direct current stimulation over the left posterior parietal cortex. *NeuroImage* 139 462–469. 10.1016/j.neuroimage.2016.06.032 27329808

[B22] Chiu Y. Dolcos F. Gonsalves B. D. Cohen N. J. (2013). On opposing effects of emotion on contextual or relational memory. *Front. Psychol.* 4:103. 10.3389/fpsyg.2013.00103 23460770 PMC3585437

[B23] Cholvin T. Loureiro M. Cassel R. Cosquer B. Herbeaux K. Vasconcelos A. D. (2016). Dorsal hippocampus and medial prefrontal cortex each contribute to the retrieval of a recent spatial memory in rats. *Brain Structure Funct.* 221 91–102. 10.1007/s00429-014-0894-6 25260556

[B24] Collins A. M. Loftus E. F. (1975). A spreading-activation theory of semantic processing. *Psychol. Rev.* 82 407–428. 10.1037/0033-295X.82.6.407

[B25] Collins A. M. Loftus E. F. (1988). A spreading-activation theory of semantic processing. *Read. Cogn. Sci.* 82 126–136. 10.1037/0033-295X.82.6.407

[B26] Córdova N. I. Turk-Browne N. B. Aly M. (2019). Focusing on what matters: modulation of the human hippocampus by relational attention. *Hippocampus* 29 1025–1037. 10.1002/hipo.23082 30779473 PMC6699927

[B27] Cruzado N. A. Tiganj Z. Brincat S. L. Miller E. K. Howard M. W. (2020). Conjunctive representation of what and when in monkey hippocampus and lateral prefrontal cortex during an associative memory task. *Hippocampus* 30 1332–1346. 10.1002/hipo.23282 33174670

[B28] Dalton M. A. Zeidman P. McCormick C. Maguire E. A. (2018). Differentiable processing of objects, associations and scenes within the hippocampus. *J. Neurosci.* 38 8146–8159. 10.1523/jneurosci.0263-18.2018 30082418 PMC6146500

[B29] Davachi L. Mitchell J. P. Wagner A. D. (2003). Multiple routes to memory: distinct medial temporal lobe processes build item and source memories. *Proc. Natl. Acad. Sci. U S A.* 100 2157–2162. 10.1073/pnas.0337195100 12578977 PMC149975

[B30] Decoteau W. E. Kesner R. P. (1998). Effects of hippocampal and parietal cortex lesions on the processing of multiple-object scenes. *Behav. Neurosci.* 112, 68–82. 10.1037/0735-7044.112.1.68 9517816

[B31] Degonda N. Mondadori C. R. Bosshardt S. Schmidt C. F. Boesiger P. Nitsch R. M. (2005). Implicit associative learning engages the hippocampus and interacts with explicit associative learning. *Neuron* 46 505–520. 10.1016/j.neuron.2005.02.030 15882649

[B32] Dixon M. L. Thiruchselvam R. Todd R. Christoff K. (2017). Emotion and the prefrontal cortex: an integrative review. *Psychol. Bull.* 143 1033–1081. 10.1037/bul0000096 28616997

[B33] Dodson C. S. Holland P. W. Shimamura A. P. (1998). Onthe recollection of specific- and partial-source information. *J. Exp. Psychol. Learn. Memory Cogn.* 24 1121–1136. 10.1037/0278-7393.24.5.1121 9747526

[B34] Duff M. C. Covington N. V. Hilverman C. Cohen N. J. (2020). Semantic memory and the hippocampus: revisiting, reaffirming, and extending the reach of their critical relationship. *Front. Hum. Neurosci.* 13:471. 10.3389/fnhum.2019.00471 32038203 PMC6993580

[B35] Dunkley B. T. Baltaretu B. Crawford J. D. (2016). Trans-saccadic interactions in human parietal and occipital cortex during the retention and comparison of object orientation. *Cortex* 82 263–276. 10.1016/j.cortex.2016.06.012 27424061

[B36] Elfman K. W. Parks C. M. Yonelinas A. P. (2008). Testing a neurocomputational model of recollection, familiarity, and source recognition. *J. Exp. Psychol. Learn. Memory Cogn.* 34 752–768. 10.1037/0278-7393.34.4.752 18605866

[B37] Fernandes N. L. Pandeirada J. N. S. Soares S. C. Nairne J. S. (2017). Adaptive memory: the mnemonic value of contamination. *Evol. Hum. Behav.* 38 451–460. 10.1016/j.evolhumbehav.2017.04.003

[B38] Foley M. A. (2018). Reflecting on how we remember the personal past: missing components in the study of memory appraisal and theoretical implications. *Memory* 26 634–652. 10.1080/09658211.2017.1387667 29035145

[B39] Ford J. H. Verfaellie M. Giovanello K. S. (2010). Neural correlates of familiarity-based associative retrieval. *Neuropsychologia* 48 3019–3025. 10.1016/j.neuropsychologia.2010.06.010 20547169 PMC2915548

[B40] Fox J. Osth A. F. (2020). Does source memory exist for unrecognized items? *PsyArXiv* [Preprint]. 10.31234/osf.io/brpwu 35084928

[B41] Fredrickson B. L. Branigan C. (2005). Positive emotions broaden the scope of attention and thought-action repertoires. *Cogn. Emot.* 19 313–332. 10.1080/02699930441000238 21852891 PMC3156609

[B42] Friedman D. (2013). The cognitive aging of episodic memory: a view based on the event-related brain potential. *Front. Behav. Neuroence* 7:111. 10.3389/fnbeh.2013.00111 23986668 PMC3752587

[B43] Fritch H. A. Macevoy S. P. Thakral P. P. Jeye B. M. Slotnick S. D. (2020). The anterior hippocampus is associated with spatial memory encoding. *Brain Res.* 1732 146696. 10.1016/j.brainres.2020.146696 32014532

[B44] Gable P. A. Harmon-Jones E. (2010). The effect of low versus high approach-motivated positive affect on memory for peripherally versus centrally presented information. *Emotion* 10 599–603. 10.1037/a0018426 20677877

[B45] Giovanello K. S. Keane M. M. Verfaellie M. (2006). The contribution of familiarity to associative memory in amnesia. *Neuropsychologia* 44 1859–1865. 10.1016/j.neuropsychologia.2006.03.004 16643967 PMC1698551

[B46] Giovanello K. S. Schnyer D. M. Verfaellie M. (2010). A critical role for the anterior hippocampus in relational memory: evidence from an fMRI study comparing associative and item recognition. *Hippocampus* 14 5–8. 10.1002/hipo.10182 15058477

[B47] Glisky E. L. Polster M. R. Routhieaux B. C. (1995). Double dissociation between item and source memory. *Neuropsychology* 9 229–235. 10.1037/0894-4105.9.2.229

[B48] Gottlieb J. (2007). From thought to action: the parietal cortex as a bridge between perception, action, and cognition. *Neuron* 53 9–16. 10.1016/j.neuron.2006.12.009 17196526

[B49] Greene N. R. Naveh-Benjamin M. (2020). A specificity principle of memory: evidence from aging and associative memory. *Psychol. Sci.* 31 316–331. 10.1177/0956797620901760 32074021

[B50] Guidotti R. Tosoni A. Perrucci M. G. Sestieri C. (2019). Choice-predictive activity in parietal cortex during source memory decisions. *NeuroImage* 189 589–600. 10.1016/j.neuroimage.2019.01.071 30708104

[B51] Guo D. Yang J. (2020). Interplay of the long axis of the hippocampus and ventromedial prefrontal cortex in schema-related memory retrieval. *Hippocampus* 30 263–277. 10.1002/hipo.23154 31490611

[B52] Harmon-Jones E. Harmon-Jones C. Amodio D. M. Gable P. A. (2011). Attitudes toward emotions. *J. Personal. Soc. Psychol.* 101 1332–1350. 10.1037/a0024951 21843012

[B53] Haskins A. L. Yonelinas A. P. Quamme J. R. Ranganath C. (2008). Perirhinal cortex supports encoding and familiarity-based recognition of novel associations. *Neuron* 59 554–560. 10.1016/j.neuron.2008.07.035 18760692

[B54] Heinke D. Humphreys G. W. (2005). “Computational models of visual selective attention: a review,” in *Connectionist Models in Psychology*, ed. Houghton G. W. (Hove: Psychological Press), 273–312.

[B55] Howard C. J. Boulton H. Bedwell S. A. Boatman C. A. Roberts K. L. Mitra S. (2019). Low-frequency repetitive transcranial magnetic stimulation to right parietal cortex disrupts perception of briefly presented stimuli. *Perception* 48 346–355. 10.1177/0301006619834251 30832537

[B56] Howard M. W. Kahana M. J. (2002). A distributed representation of temporal context. *J. Mathemat. Psychol.* 46 269–299. 10.1006/jmps.2001.1388

[B57] Huntsinger J. R. (2013). Does emotion directly tune the scope of attention? *Curr. Direct. Psychol. Sci.* 22 265–270. 10.1177/0963721413480364

[B58] Iwasaki S. Sasaki T. Ikegaya Y. (2021). Hippocampal beta oscillations predict mouse object-location associative memory performance. *Hippocampus* 31 503–511. 10.1002/hipo.23311 33556218

[B59] Jacoby L. L. (1991). A process dissociation framework: Separating automatic from intentional uses of memory. *J. Memory Lang.* 30 513–541. 10.1016/0749-596X(91)90025-F

[B60] Jacoby L. L. Bishara A. J. Hessels S. Toth J. P. (2005). Aging, subjective experience, and cognitive control:dramatic false remembering by older adults. *J. Exp. Psychol. General* 134 131–148. 10.1037/0096-3445.134.2.131 15869342

[B61] Johnson M. K. (2005). The relation between source memory and episodic memory: comment on Siedlecki et al. (2005). *Psychol. Aging* 20 529–531. 10.1037/0882-7974.20.3.529 16248712

[B62] Johnson M. K. Hashtroudi S. Lindsay D. S. (1993). Source monitoring. *Psychol. Bull.* 114 3–28. 10.1037/0033-2909.114.1.3 8346328

[B63] Kahneman D. Treisman A. Gibbs B. J. (1992). The reviewing of object files: object-specific integration of information. *Cogn. Psychol.* 24 175–219. 10.1016/0010-0285(92)90007-O 1582172

[B64] Kalisch R. Wiech K. Critchley H. D. Dolan R. J. (2006). Levels of appraisal: a medial prefrontal role in high-level appraisal of emotional material. *NeuroImage* 30 1458–1466. 10.1016/j.neuroimage.2005.11.011 16388969

[B65] Kaplan R. L. Ilse V. D. Levine L. J. (2012). Motivation matters: differing effects of pre-goal and post-goal emotions on attention and memory. *Front. Psychol.* 3:404. 10.3389/fpsyg.2012.00404 23162490 PMC3498897

[B66] Katsuki F. Saito M. Constantinidis C. (2015). Influence of monkey dorsolateral prefrontal and posterior parietal activity on behavioral choice during attention tasks. *Eur. J. Neurosci.* 40 2910–2921. 10.1111/ejn.12662 24964224 PMC4172489

[B67] Kensinger E. A. Corkin S. (2008). “Amnesia: point and counterpoint,” in *Learning Theory and Behavior. Learning and Memory: A Comprehensive Reference*, Vol. 1 eds Byrne J. Menzel R. (Amsterdam: Elsevier), 259–286. 10.1007/BF00157784

[B68] Kensinger E. A. Garoff-Eaton R. J. Schacter D. L. (2007). Effects of emotion on memory specificity: memory trade-offs elicited by negative visually arousing stimuli. *J. Memory Lang.* 56 575–591. 10.1016/j.jml.2006.05.004

[B69] Kesner R. P. (2000). Behavioral analysis of the contribution of the hippocampus and parietal cortex to the processing of information: interactions and dissociations. *Hippocampus* 10, 483–490. 10.1002/1098-1063(2000)10:43.0.CO;2-Z 10985288

[B70] Kim K. Yi D. Johnson M. K. (2012). Negative effects of item repetition on source memory. *Memory Cogn.* 40 889–901. 10.3758/s13421-012-0196-2 22411165 PMC4922421

[B71] King D. R. Miller M. B. (2017). Influence of response bias and internal/external source on lateral posterior parietal successful retrieval activity. *Cortex* 91 126–141. 10.1016/j.cortex.2017.04.002 28499558

[B72] Kinjo H. (2011). Effects of aging and divided attention on recognition memory processes for single and associative information. *Psychol. Rep.* 108 405–419. 10.2466/04.10.22.PR0.108.2.405-419 21675557

[B73] Klauer K. C. Wegener I. (1998). Unraveling socialcategorization in the “Who said What?” paradigm. *J. Personal. Soc. Psychol.* 75 1155–1178. 10.1037/0022-3514.75.5.1155 9866182

[B74] Kroneisen M. Bell R. (2013). Sex, cheating, and disgust: enhanced source memory for trait information that violates gender stereotypes. *Memory* 21 167–181. 10.1080/09658211.2012.713971 22928947

[B75] Kroneisen M. Bell R. (2018). Remembering the place with the tiger: survival processing can enhance source memory. *Psychonomic Bull. Rev.* 25 667–673. 10.3758/s13423-018-1431-z 29464521

[B76] Leshikar E. D. Duarte A. (2012). Medial prefrontal cortex supports source memory accuracy for self-referenced items. *Soc. Neurosci.* 7 126–145. 21936739 10.1080/17470919.2011.585242 PMC3701388

[B77] Levine L. J. Edelstein R. S. (2009). Emotion and memory narrowing: a review and goal-relevance approach. *Cogn. Emot.* 23 833–875. 10.1080/02699930902738863

[B78] Levita L. Muzzio I. A. (2010). Role of the hippocampus in goal-oriented tasks requiring retrieval of spatial versus non-spatial information. *Neurobiol. Learn. Mem.* 93 581–588. 10.1016/j.nlm.2010.02.006 20206279

[B79] Lewis P. A. Critchley H. D. (2003). Mood-dependent memory. *Trends Cogn. Sci.* 7 431–433. 10.1016/j.tics.2003.08.005 14550485

[B80] Li S. C. Naveh-Benjamin M. Lindenberger U. (2005). Aging neuromodulation impairs associative binding neurocomputational account. *Psychol. Sci.* 16 445–450. 10.1111/j.0956-7976.2005.01555.x 15943670

[B81] Logan G. D. (1996). The code theory of visual attention: an integration of space-based and object-based attention. *Psychol. Rev.* 103 603–649. 10.1037/0033-295X.103.4.603 8888649

[B82] Ma S. T. Abelson J. L. Okada G. Taylor S. F. Liberzon I. (2017). Neural circuitry of emotion regulation: effects of appraisal, attention, and cortisol administration. *Cogn. Affect. Behav. Neurosci.* 17 437–451. 10.3758/s13415-016-0489-1 28032303

[B83] Madan C. R. Fujiwara E. Caplan J. B. Sommer T. (2017). Emotional arousal impairs association-memory: roles of amygdala and hippocampus. *NeuroImage* 156 14–28. 10.1016/j.neuroimage.2017.04.065 28483720

[B84] Madan C. R. Scott S. M. E. Kensinger E. A. (2019). Positive emotion enhances association-memory. *Emotion* 19 733–740. 10.1037/emo0000465 30124317 PMC6612425

[B85] Manns J. R. Hopkins R. O. Squire L. R. (2003). Semantic memory and the human hippocampus. *Neuron* 38 127–133. 10.1016/S0896-6273(03)00146-6 12691670

[B86] Manns J. R. Howard M. W. Eichenbaum H. (2007). Gradual changes in hippocampal activity support remembering the order of events. *Neuron* 56 530–540. 10.1016/j.neuron.2007.08.017 17988635 PMC2104541

[B87] Maratos E. J. Dolan R. J. Morris J. S. Henson R. N. Rugg M. D. (2001). Neural activity associated with episodic memory for emotional context. *Neuropsychologia* 39 910–920. 10.1016/s0028-3932(01)00025-2 11516444

[B88] Maratos E. J. Rugg M. D. (2011). Electrophysiological correlates of the retrieval of emotional and non-emotional context. *J. Cogn. Neurosci.* 13 877–891. 10.1162/089892901753165809 11595092

[B89] Mather M. (2007). Emotional arousal and memory binding: an object-based framework. *Perspect. Psychol. Sci.* 2 33–52. 10.1111/j.1745-6916.2007.00028.x 26151918

[B90] Mather M. Clewett d. Sakaki M. Harley C. W. (2016). Norepinephrine ignites local hotspots of neuronal excitation: how arousal amplifies selectivity in perception and memory. *Behav. Brain Sci.* 39. 10.1017/S0140525X15000667 26126507 PMC5830137

[B91] Mather M. Mitchell K. J. Raye C. L. Novak D. Greene E. J. Johnson M. K. (2006). Emotional arousal can impair feature binding in working memory. *J. Cogn. Neurosci.* 18 614–625. 10.1162/jocn.2006.18.4.614 16768364

[B92] Mechias M. Etkin A. Kalisch R. (2009). A meta-analysis of instructed fear studies: Implications for conscious appraisal of threat. *NeuroImage* 49 1760–1768. 10.1016/j.neuroimage.2009.09.040 19786103

[B93] Medina J. Jax S. A. Coslett H. B. (2020). Impairments in action and perception after right intraparietal damage. *Cortex* 122 288–299. 10.1016/j.cortex.2019.02.004 30879643

[B94] Meiser T. Bröder A. (2002). Memory for multidimensional source information. *J. Exp. Psychol. Learn. Memory Cogn.* 28 116–137. 10.1037//0278-7393.28.1.116 11827074

[B95] Milad M. R. Wright C. Orr S. P. Pitman R. K. Quirk G. J. Rauch S. L. (2007). Recall of fear extinction in humans activates the ventromedial prefrontal cortex and hippocampus in concert. *Biol. Psychiatry* 62 446–454. 10.1016/j.biopsych.2006.10.011 17217927

[B96] Mitchell K. J. Johnson M. K. (2009). Source monitoring 15 years later: what have we learned from fMRI about the neural mechanisms of source memory? *Psychol. Bull.* 135 638–677. 10.1037/a0015849 19586165 PMC2859897

[B97] Mogg K. Bradley B. P. (1998). A cognitive-motivational analysis of anxiety. *Behav. Therapy* 36 809–848. 10.1016/S0005-7967(98)00063-1 9701859

[B98] Müri R. Iba-Zizen M. T. Derosier C. Cabanis E. A. Pierrot-Deseilligny C. (1996). Location of the human posterior eye field with functional magnetic resonance imaging. *J. Neurol. Neurosurg. Psychiatry* 60 445–448. 10.1136/jnnp.60.4.445 8774415 PMC1073903

[B99] Nairne J. S. Pandeirada J. N. S. Gregory K. J. Van Arsdall J. E. (2009). Adaptive memory: fitness relevance and the hunter-gatherer mind. *Psychol. Sci.* 20 470–476. 10.1111/j.1467-9280.2009.02356.x 19422622

[B100] Nairne J. S. Vanarsdall J. E. Pandeirada J. Blunt J. R. (2012). Adaptive memory: enhanced location memory after survival processing. *J. Exp. Psychol. Learn. Memory Cogn.* 38 495–501. 10.1037/a0025728 22004268

[B101] Nie A. (2018). Facial recall: feature–conjunction effects in source retrieval versus item recognition. *Percept. Mot. Skills* 125 369–386. 29307254 10.1177/0031512517751725

[B102] Nordin K. Herlitz A. Larsson E. M. Söderlund H. (2017). Overlapping effects of age on associative memory and the anterior hippocampus from middle to older age. *Behav. Brain Res.* 317 350–359. 10.1016/j.bbr.2016.10.002 27713000

[B103] Nyberg L. Marklund P. Persson J. Cabeza R. Ingvar M. (2003). Common prefrontal activations during working memory, episodic memory, and semantic memory. *Neuropsychologia* 41 371–377. 10.1016/S0028-3932(02)00168-9 12457761

[B104] Onyper S. V. Zhang Y. X. Howard M. W. (2010). Some-or-none recollection: evidence from item and source memory. *J. Exp. Psychol. General* 139 341–364. 10.1037/a0018926 20438255 PMC2864935

[B105] Osth A. F. Fox J. McKague M. Heathcote A. Dennis S. (2018). The list strength effect in source memory: data and a global matching model. *J. Memory Lang.* 103 91–113. 10.31219/osf.io/ky9ax

[B106] Öztekin I. McElree B. Staresina B. P. Davachi L. (2009). Working memory retrieval: contributions of the left prefrontal cortex, the left posterior parietal cortex, and the hippocampus. *J. Cogn. Neurosci.* 21 581–593. 10.1162/jocn.2008.21016 18471055 PMC2778249

[B107] Parks C. M. Yonelinas A. P. (2015). The importance of unitization for familiarity-based learning. *J. Exp. Psychol. Learn. Memory Cogn.* 41 881–903. 10.1037/xlm0000068 25329077 PMC4404176

[B108] Pergollzzi D. Chua E. F. (2017). Transcranial direct current stimulation over the parietal cortex alters bias in item and source memory tasks. *Brain Cogn.* 108 56–65. 10.1016/j.bandc.2016.06.009 27474794 PMC5014655

[B109] Polyn S. M. Norman K. A. Kahana M. J. (2009). A context maintenance and retrieval model of organizational processes in free recall. *Psychol. Rev.* 116 129–156. 10.1037/a0014420 19159151 PMC2630591

[B110] Preston A. Eichenbaum H. (2013). Interplay of hippocampus and prefrontal cortex in memory. *Curr. Biol.* 23 764–773. 10.1016/j.cub.2013.05.041 24028960 PMC3789138

[B111] Raaijmakers J. G. W. Schiffrin R. (1981). Search of associative memory. *Psychol. Rev.* 8 98–134. 10.1037/0033-295X.88.2.93

[B112] Ragland J. D. Laird A. R. Ranganath C. Blumenfeld R. S. Glahn D. C. (2009). Prefrontal activation deficits during episodic memory in schizophrenia. *Am. J. Psychiatry* 166 863–874. 10.1176/appi.ajp.2009.08091307 19411370 PMC2885958

[B113] Reyna V. F. (2000). Fuzzy-trace theory and source monitoring: an evaluation of theory and false-memory data. *Learn. Individual Diff.* 12 163–175. 10.1016/S1041-6080(01)00034-6

[B114] Rhodes S. M. Donaldson D. I. (2008). Electrophysiological evidence for the effect of interactive imagery on episodic memory: encouraging familiarity for non-unitized stimuli during associative recognition. *NeuroImage* 39 873–884. 10.1016/j.neuroimage.2007.08.041 17950624

[B115] Riefer D. M. Hu X. Batchelder W. H. (1994). Response strategies in source monitoring. *J. Exp. Psychol. Learn. Memory Cogn.* 20 680–693. 10.1037/0278-7393.20.3.680

[B116] Ries M. L. Mclaren D. G. Bendlin B. B. Xu G. Rowley H. A. Birn R. (2012). Medial prefrontal functional connectivity—relation to memory self-appraisal accuracy in older adults with and without memory disorders. *Neuropsychologia* 50 603–611. 10.1016/j.neuropsychologia.2011.12.014 22230228 PMC3537182

[B117] Ro T. Wallace R. Hagedorn J. Farnè A. Pienkos E. (2004). Visual enhancing of tactile perception in the posterior parietal cortex. *J. Cogn. Neurosci.* 16 24–30. 10.1162/089892904322755520 15006033

[B118] Roozendaal B. McEwen B. S. Chattarji S. (2009). Stress, memory and the amygdala. *Nat. Rev. Neurosci.* 10 423–433. 10.1038/nrn2651 19469026

[B119] Sahakyan L. Kelley C. M. (2002). A contextual change account of the directed forgetting effect. *J. Exp. Psychol. Learn. Memory Cogn.* 28 1064–1072. 10.1037/0278-7393.28.6.1064 12450332

[B120] Schacter D. L. Kaszniak A. W. Kihlstrom J. F. Valdiserri M. (1991). The relation between source memory and aging. *Psychol. Aging* 6 559–568. 10.1037/0882-7974.6.4.559 1777144

[B121] Sederberg P. B. Howard M. W. Kahana M. J. (2008). A context-based theory of recency and contiguity in free recall. *Psychol. Rev.* 115 893–912. 10.1037/a0013396 18954208 PMC2585999

[B122] Slotnick S. D. (2010). ‘Remember’ source memory rocs indicate recollection is a continuous process. *Memory* 18 27–39. 10.1080/09658210903390061 19937493

[B123] Slotnick S. D. Dodson C. S. (2005). Support for a continuous (single-process) model of recognition memory and source memory. *Memory Cogn.* 33 151–170. 10.3758/BF03195305 15915801

[B124] Slotnick S. D. Moo L. R. Segal J. B. Hart J. Jr. (2003). Distinct prefrontal cortex activity associated with item memory and source memory for visual shapes. *Cogn. Brain Res.* 17 75–82. 10.1016/S0926-6410(03)00082-X 12763194

[B125] Snytte J. Elshiekh A. Subramaniapillai S. Manning L. Rajah M. N. (2020). The ratio of posterior–anterior medial temporal lobe volumes predicts source memory performance in healthy young adults. *Hippocampus* 30 1209–1227. 10.1002/hipo.23251 32830426

[B126] Sofia P. Gregoriou G. G. (2017). Top-down control of visual attention by the prefrontal cortex: functional specialization and long-range interactions. *Front. Neurosci.* 11:545. 10.3389/fnins.2017.00545 29033784 PMC5626849

[B127] Squire L. R. (2004). Memory systems of the brain: a brief history and current perspective. *Neurobiol. Learn. Mem.* 82 171–177. 10.1016/j.nlm.2004.06.005 15464402

[B128] Starns J. J. Ksander J. C. (2016). Item strength influences source confidence and alters source memory zROC slopes. *J. Exp. Psychol. Learn. Memory Cogn.* 42 351–365. 10.1037/xlm0000177 26371494

[B129] Starns J. J. Pazzaglia A. M. Rotello C. M. Hautus M. J. Macmillan N. A. (2013). Unequal-strength source zROC slopes reflect criteria placement and not (necessarily) memory processes. *J. Exp. Psychol. Learn. Memory Cogn.* 39 1377–1392. 10.1037/a0032328 23565789 PMC3896244

[B130] Steinberger A. Payne J. D. Kensinger E. A. (2011). The effect of cognitive reappraisal on the emotional memory trade-off. *Cogn. Emot.* 25 1237–1245. 10.1080/02699931.2010.538373 21432629 PMC3637931

[B131] Sutton S. K. Davidson R. J. (1997). Resting prefrontal asymmetry: a biological substrate of the behavioral approach and behavioral inhibition system. *Psychol. Sci.* 8, 204–210. 10.1111/j.1467-9280.1997.tb00413.x

[B132] Swick D. Senkfor A. Petten C. V. (2006). Source memory retrieval is affected by aging and prefrontal lesions: behavioral and ERP evidence. *Brain Res.* 1107 161–176. 10.1016/j.brainres.2006.06.013 16828722 PMC2365725

[B133] Takehara-Nishiuchi K. (2020). Prefrontal–hippocampal interaction during the encoding of new memories. *Brain Neurosci. Adv.* 4:2398212820925580. 10.1177/2398212820925580 32954000 PMC7479858

[B134] Talmi D. Lohnas L. J. Daw N. D. (2019). A retrieved context model of the emotional modulation of memory. *Psychol. Rev.* 126 455–485. 10.1101/175653 30973247

[B135] Talmi D. Luk B. T. C. McGarry L. M. Moscovitch M. (2007). The contribution of relatedness and distinctiveness to emotionally-enhanced memory. *J. Memory Lang.* 56 555–574. 10.1016/j.jml.2007.01.002

[B136] Talmi D. Moscovitch M. (2004). Can semantic relatedness explain the enhancement of memory for emotional words? *Memory Cogn.* 32 742–751. 10.3758/BF03195864 15552351

[B137] Tulving E. (2002). Episodic memory: from mind to brain. *Annu. Rev. Psychol.* 53 1–25. 10.1146/annurev.psych.53.100901.135114 11752477

[B138] Tulving E. (2004). Episodic memory: from mind to brain. *Annu. Rev. Psychol.* 53 1–25. 10.1146/annurev.psych.53.100901.135114 11752477

[B139] Tulving E. Kapur S. Craik F. Houle M. S. (1994). Hemispheric encoding/retrieval asymmetry in episodic memory: positron emission tomography findings. *Proc. Natl. Acad. Sci. U. S. A.* 91 2016–2020. 10.1073/pnas.91.6.2016 8134342 PMC43300

[B140] Tulving E. Thomson D. M. (1973). Encoding specificity and retrieval processes in episodic memory. *Psychol. Rev.* 80 352–373. 10.1037/h0020071

[B141] Turi G. F. Li W. Chalis S. Pandi I. O’Hare J. Priestley J. B. (2019). Vasoactive intestinal polypeptide-expressing interneurons in the hippocampus support goal-oriented spatial learning. *Neuron* 101 1150–1165. 10.1016/j.neuron.2019.01.009 30713030 PMC6428605

[B142] Vargha-Khadem F. (1997). Differential effects of early hippocampal pathology on episodic and semantic memory. *Science* 277 376–380. 10.1126/science.277.5324.376 9219696

[B143] Venneri A. Mitolo M. Beltrachini L. Varma S. Pietà C. D. Jahn-Carta C. (2019). Beyond episodic memory: semantic processing as independent predictor of hippocampal/perirhinal volume in aging and mild cognitive impairment due to Alzheimer’s disease. *Neuropsychology* 33 523–533. 10.1037/neu0000534 30777767

[B144] von Stein A. Rappelsberger P. Sarnthein J. Petsche H. (1999). Synchronization between temporal and parietal cortex during multimodal object processing in man. *Cerebral Cortex* 9, 137–150. 10.1093/cercor/9.2.137 10220226

[B145] Vuli K. Bjeki J. Paunovi D. Jovanovi M. Filipovi S. R. (2021). Theta-modulated oscillatory transcranial direct current stimulation over posterior parietal cortex improves associative memory. *Sci. Rep.* 11:3013. 10.1038/s41598-021-82577-7 33542344 PMC7862221

[B146] Woodward A. (1998). Infants selectively encode the goal object of an actor’s reach. *Cognition* 69 1–34. 10.1016/S0010-0277(98)00058-4 9871370

[B147] Yonelinas A. P. (1999). The contribution of recollection and familiarity to recognition and source-memory judgments: a formal dual-process model and an analysis of receiver operating characteristics. *J. Exp. Psychol. Learn. Memory Cogn.* 25 1415–1434. 10.1037/0278-7393.25.6.1415 10605829

[B148] Zhang Q. Liu X. An W. Yang Y. Wang Y. (2015). Recognition memory of neutral words can be impaired by task-irrelevant emotional encoding contexts: behavioral and electrophysiological evidence. *Front. Hum. Neurosci.* 9:73. 10.3389/fnhum.2015.00073 25762916 PMC4327741

[B149] Zheng Z. Li J. Xiao F. Ren W. He R. (2016). Unitization improves source memory in older adults: an event-related potential study. *Neuropsychologia* 89 232–244. 10.1016/j.neuropsychologia.2016.06.025 27343684

